# Forkhead Box Transcription Factor (FOXO3a) mediates the cytotoxic effect of vernodalin in vitro and inhibits the breast tumor growth in vivo

**DOI:** 10.1186/s13046-015-0266-y

**Published:** 2015-12-08

**Authors:** Suresh Kumar Ananda Sadagopan, Nooshin Mohebali, Chung Yeng Looi, Mohadeseh Hasanpourghadi, Ashok Kumar Pandurangan, Aditya Arya, Hamed Karimian, Mohd Rais Mustafa

**Affiliations:** Department of Pharmacology, Faculty of Medicine, University of Malaya, Kuala Lumpur, 50603 Malaysia; Department of Biochemistry, Central Leather Research Institute, Council of Scientific and Industrial Research (CSIR), Adyar, Chennai, 600 020 India; Department of Pharmacy, Faculty of Medicine, University of Malaya, Kuala Lumpur, 50603 Malaysia

**Keywords:** Breast cancer, Vernodalin, Cancer prevention, FOXO3a, Akt

## Abstract

**Background:**

Natural compounds have been demonstrated to lower breast cancer risk and sensitize tumor cells to anticancer therapies. Recently, we demonstrated that vernodalin (the active constituent of the medicinal herb *Centratherum anthelminticum* seeds) induces apoptosis in breast cancer cell-lines. The aim of this work was to gain an insight into the underlying anticancer mechanism of vernodalin using in vitro and in vivo model.

**Methods:**

Vernodalin was isolated through the bioassay guided fractionation from the seeds. The protein expression of p-Akt, PI3K, FOXO3a, Bim, p27kip1, cyclinD1, and cyclinE was examined by the Western blot analysis. Immunoprecipitation assays were performed to analyse Akt kinase activity. Small interfering RNA (siRNA) was used to study the role of FOXO3a upregulation and their targets during vernodalin treatment. Immunofluorescence, subcellular localisation of FOXO3a by Western blot was performed to analyse FOXO3a localisation in nucleus of breast cancer cells. Immunohistochemical analysis of PCNA, Ki67, p27kip1, FOXO3a and p-FOXO3a in the LA7-induced mammary gland tumor model was performed.

**Results:**

Our results showed that vernodalin regulates cancer cell apoptosis through activation of FOXO transcription factors and its downstream targets (Bim, p27Kip1, p21Waf1/cip1, cyclin D1, cyclin E) as examined by Western blots. Furthermore, we showed that FOXO3a/PI3K-Akt played a significant role in vernodalin induced apoptosis in breast cancer cells. Immunoprecipitation assays showed Akt kinase activity was downregulated. Immunofluorescence, subcellular fractionation and Western blot showed FOXO3a accumulation in the nucleus of breast cancer cells after vernodalin treatment. Silencing of FOXO3a protected breast cancer cells against vernodalin induced apoptosis. The anti-tumor action of vernodalin was further confirmed by examining cell proliferative markers, PCNA and Ki67 in the LA7-induced mammary gland tumor model. We also corroborated our findings in vivo by showing upregulation of p27Kip1, FOXO3a and decrease in the p-FOXO3a level in vernodalin-treated breast tumor tissue.

**Conclusions:**

Our results suggest that PI3K-Akt/FOXOa pathway is a critical mediator of vernodalin-induced cytotoxicity and this compound could be further developed as a potential chemopreventive or chemotherapeutic agent for breast cancer therapy.

**Electronic supplementary material:**

The online version of this article (doi:10.1186/s13046-015-0266-y) contains supplementary material, which is available to authorized users.

## Background

Breast cancer is the third leading cause of cancer deaths and highest among women aged between 20 and 59 years based on a cancer statistics in 2013 [1]. It is still the leading cause of morbidity and mortality of women worldwide; with 23 % (1.38 million) of new cancer cases and 14 % (458,400) of the total cancer deaths [2]. Breast cancer development and metastasis is a multistep process, often caused by dysfunction of several regulatory features those keep cells in check [3]. Continents such as North America, Western Europe and Australia have been reported to have the highest incidence rates. Nevertheless, it is increasing in all parts of the globe, especially in the developing countries. Steroid hormones, particularly estrogen, contribute to the development and progression of mammary gland neoplasia. Majority of breast cancer is sporadic and the risk factors are mostly linked to estrogen exposure and age [4]. The outcome of breast cancer treatment during the past several years has significant improvement, primarily due to early diagnosis and chemotherapies. However, there is a relative lack of effective therapies for advanced-stage metastatic disease [5].

Most of the chemo-therapeutic drugs in market for treating breast cancer cause harmful effects such as severe nausea, vomiting, fatigue and hair loss. Hence, there is an urgent need to develop alternative therapeutic drugs with negligible side effects. One such alternative could be the use of plant based chemo-therapeutic drugs. Natural products, including phytoconstituents and dietary agents have already been shown to suppress the growth of breast tumor cells through modulation of cell proliferation, cellular differentiation, apoptosis, angiogenesis as well as several signal transduction pathways [6, 7]. Certain natural products are used in cancer chemo-prevention to inhibit or revert carcinogenesis and to suppress cancer malignancy [8]. The efficacy of several dietary supplements and natural products in breast cancer has been tested by clinical intervention trials that support these potential utility of these agents in the breast cancer prevention, treatment, and management regimens in the future [9]. In addition, epidemiological data suggest that consumption of plant foods or natural products (47.2 %) and botanical supplements (47.5 %) that contain high levels of antioxidants can slow down or prevent the appearance of cancer [10, 11].

ForkheadboxO (FOXO) proteins, a subgroup of the forkhead transcription factors, play an important role in tumor suppression mainly by upregulating target genes involved in cell cycle arrest and apoptosis [12]. In mammals, this family of proteins consists of four members- FOXO1, FOXO3, FOXO4 and FOXO6, and accumulated evidence shows that FOXO3a regulates a wide range of biological processes, including proliferation, apoptosis, protection against oxidative stress, and metabolism [13]. In particular one of the FOXO members, FOXO3a is under-expressed in many breast cancer patients [14]. Low levels of FOXO3 have been reported to confer chemotherapy resistance in human cancers, being significantly associated with poor prognosis in cancer patients [15, 16]. FOXO3a regulation and activity depends mainly on the subcellular localisation, which can be achieved via post-translational modifications including phosphorylation, acetylation and ubiquitination [17]. Several studies indicate that the FOXO family is a key downstream target of PI3K-Akt pathway in development and longevity [18, 19]. Akt controls transcription of FOXO3a target genes, through modulation of FOXO3a activity by phosphorylating three conserved threonine/serine residues (Thr-32, Ser-253, and Ser-315), leading to the release of FOXO3a from DNA and translocation to the cytoplasm [20, 21]. Reports suggest that up regulation of FOXO3a proteins can promote apoptosis through upregulation of the pro-apoptotic Bcl-2 family member (Bim) and consequent increase in the cell cycle protein levels of p27kip1. In contrast a CDK inhibitor, (CKI) is capable of associating with cyclin-CDK complexes and inhibits the apoptosis [22–24]. Therefore, FOXO3a has been proposed as an important factor influencing the efficacy of a variety of chemotherapeutic drugs. In fact, the restoration of FOXO3a expression has been exploited for some mechanism-based anticancer therapies [25, 26].

Cells replicate their genome and divide by the cell cycle process. During this process, the cell cycle control is a cyclically operating biochemical device consisted of numerous interacting proteins, which induce and organize proper progression, including cyclins, cyclin-dependent kinases (CDK) and their inhibitors (CDKI) [27, 28]. Dysregulation of these regulators could give rise to cell growth uncontrollably. PI3K/Akt signaling pathway is a vital transduction pathway in regulating cell cycle and apoptosis. Different types of cancer, including human breast cancer and lung cancer, reportedly activate this pathway [29]. Recent studies have shown that some anti-cancer drugs could down-regulate Akt to induce G1/S arrest [30, 31]. Overactive PI3K/Akt pathway decreases apoptosis and tumor cell cycle arrest, which leads to growth or proliferation of cancer. Akt phosphorylates GSK3β and blocks its kinase activity, thereby allowing cyclin D1 to accumulate which could inhibit cells into S period from G1 phase. Akt can also negatively influence the expression of cell cycle inhibitors such as KIP1 (also known as p27) and WAF1 (also known as CIP1 or p21) [32].

*Centratherum anthelminticum* (L.) Kuntze, (plant name has been confirmed with www.theplantlist.org) generally known as kalajiri, somraj, black cumin or bitter cumin, is a member of Asteraceae family. The seeds of *C. anthelminticum* have been reported to have various pharmacological properties, such as anti-viral, anti-filarial, anti-microbial, anti-fungal and anti-diabetic activities [33–35]. We recently reported that the chloroform fraction of *C. anthelminticum* seeds exhibited anti-oxidant property by inhibiting tumor necrosis factor-α, and inhibiting breast cancer cells growth by interrupting the activation of nuclear factor-kappa B [36]. Our earlier report demonstrated that vernodalin (the active compound isolated through bioassay guided fractionation) can induce apoptosis in human breast cancer cells via caspase pathway [37]. Vernodalin (colourless oil) is a cytotoxic sesquiterpene lactone having two active α, β-unsaturated enoate moieties (one on the lactone ring and the other on the side chain). However, the molecular aspect of vernodalin and its in vivo anti-tumor efficacy have not been studied. This present study seek to investigate the cellular mechanisms underlying the cytotoxicity effect of vernodalin in human breast cancer cell lines and rat LA7 induced mammary gland tumor model. Our results suggest that FOXO3a is a key mediator of vernodalin induced cell cycle arrest and apoptosis in breast cancer cells.

## Methods

### Plant material

The seeds of *C. anthelminticum* were procured from the medicinal plant cultivation zone of Amritum Bio-Botanica Herbs Research Laboratory Pvt. Ltd, Betul Madhya Pradesh India. Voucher specimen (CA-9) was deposited (9^th^ August 2008) in the Department of Pharmacology, University Malaya.

### Extraction and isolation

The powdered seeds of *C. anthelminticum* (150 g) were extracted successively with chloroform (CHCl_3_) (3X250ml) (Merck, Darmstadt, Germany) in a Soxhlet apparatus for 24 h. The extracts were collected, filtered and concentrated to dryness under reduced pressure in a rotary evaporator (<40 °C) under vacuum to yield 8.53 g. Then the dried samples were fractionated into six fractions using High Performance Liquid Chromatography (HPLC) as described previously [37]. The chemical structure of vernodalin was shown in the Additional file 1: Figure S1. The fraction containing vernodalin were further subjected to HPLC isolation until >90 % pure. The purified compound, vernodalin was confirmed through spectral analysis as done in our previous publication [37].

### Cell culture

The human breast cancer cell lines, (MCF-7; MDA-MB 231) and rat mammary tumor cell line (LA7) were purchased from the American Type Culture Collection (ATCC, Manassas, VA, USA). Cells were cultured in Dulbecco’s Modified Eagle Medium (Gibco BRL, Carlsbad, CA, USA) supplemented with 10 % heat-inactivated fetal bovine serum, 2 mM glutamine, 1 % penicillin and streptomycin. LA7 cells were maintained in Dulbecco’s Modified Eagle’s Medium supplemented with 5 % fetal bovine serum, 1 % penicillin and streptomycin, 4.5 g/L glucose, 0.005 mg/mL insulin, and 20 mM HEPES in humidified environment with 5 % CO_2_ at 37 °C. For experimental purposes, cells in exponential growth phase (approximately 70-80 % confluency) were used.

### Immunofluorescence analysis of FOXO3

1X10^4^ cells per well were seeded onto 96-well plate. Cells were treated with vernodalin or DMSO (negative control) and Doxorubicin (Purchased from Pfizer, USA) at indicated concentrations for 24 h. The vernodalin concentration and time was fixed based upon our previous publication [37]. Cells were fixed with 4 % formaldehyde for 15 min. Fixed cells were permeabilized with 0.1 % Triton X-100 in phosphate buffer saline (PBS). Samples were blocked with 3 % bovine serum albumin and incubated with FOXO3a primary rabbit antibody (Cell Signaling Technology, Danvers, MA) for 1 h. Samples were washed three times with wash buffer I (1 X PBS) before addition of goat anti rabbit secondary antibodies conjugated with DyLight™ 488 (Thermo Scientific). Cells were rinsed three times with wash buffer II (1 X PBS with 1 % Tween-20). Nucleus was stained with Hoechst 33258. Stained cells were visualized and images were captured using Cellomics ArrayScan HCS reader (Thermo Scientific, USA). Cell health profiling bioapplication module was used to quantify the fluorescence intensities of each dye.

### Protein extraction

Cells treated with or without vernodalin for 24 h were collected and lysed in cell lysis buffer (1 % NP-40, 0.5 % sodium deoxycholate, 0.1 % SDS) supplemented with freshly added 10 mM β-glycerophosphate, 1 mM sodium orthovanadate, 10 mM NaF, 1 mM phenylmethylsulfonyl fluoride and Protease Inhibitor Cocktail (Santa Cruz, CA) for 30 min on ice. Then lysates were centrifuged at 12,000 × g for 30 min at 4 °C. The concentration of total protein was determined by Bradford assay (Bio-Rad, Hercules, CA). The protein extracts were stored at -80 °C until further processing.

### Westernblot analysis

The extracts are heated in a boiling water bath for 5 min and equal amounts of protein (40 μg) were separated by 10 % polyacrylamide gel. Proteins were then transferred to microporous polyvinylidene difluoride (PVDF) membrane (Milipore). Membranes were incubated in 5 % BSA (Sigma) blocking buffer for 1 h at room temperature. Incubations with primary antibody were carried out overnight at 4 °C. Immunoblotting was performed with the following antibodies: anti-FOXO3a, anti-p-FOXO3a (Ser-253), anti-p21Cip1/waf1, anti- p27Kip1, anti-Bim, anti-cyclinD1, anti-cyclinE, anti-Akt (total), anti-p-Akt (Thr-308), anti-p-PI3K, anti-total-PI3K, anti-GAPDH, anti-LaminB1 (1:1000) (Cell Signaling Technology, Danvers, MA) and mouse anti-β-actin (1:10000) (Sigma) antibodies overnight at 4 °C. The next day, the membranes were washed three times for 10 min in TBS-T and incubated with the corresponding horseradish peroxidase-conjugated secondary antibody for 1 h. To remove excess antibodies, membranes were washed 4 times before HRP activities were detected using ECL Plus Chemiluminescence Reagent (Amersham, Chalfont, UK) according to the protocol supplied with the kit. Finally the quantification was done by ImageJ software (NIH, Bethesda, MD).

### Akt kinase activity

After appropriate treatments, Akt kinase assay was performed using a non-radioactive Akt kinase assay kit (Cell Signaling Technology) following the manufacturer’s instructions. Briefly, Akt was immunoprecipitated from 100 μg cell lysate overnight using an immobilized Akt antibody. The beads were then pelleted and washed twice in 500 μL of cell lysis buffer and twice in 500 μL of kinase buffer. Pellets were resuspended in 25 μL of kinase buffer containing 0.5 mL of 10 mM ATP, 1 mg Glycogen synthase kinase-3 (GSK-3) fusion protein for 30 min at 30 °C. Kinase reaction was terminated by adding sample buffer containing β-mercapatoethanol. Finally, the activity of Akt kinase in each sample was determined according to GSK-3α/β phosphorylation by Western blot.

### Preparation of cytosol/nuclear extract

At 24 h after treatment, the MCF-7 and MDA- MB 231 cells were harvested and washed 3 times with cold phosphate-buffered saline (PBS). The cytoplasmic and nuclear protein fractions were extracted using NE-PER nuclear/cytoplasmic extraction reagent (Cell Signalling Technology). In brief 5 × 10^6^ cells is pelleted by centrifugation at 500 × g for 2-3 min. The cell pellet were treated with ice-cold 500 μl of CER I (cytoplasmic extraction reagent 1) then vortex the tube vigorously on the highest setting for 15 s to fully suspend the cell pellet. Incubate the tube on ice for 10 min then add ice-cold 27.5 μl of CER II to the tube then vortex the tube for 5 s on the highest setting. Incubate tube on ice for 1 min; centrifuge the tube for 5 min at maximum speed in a microcentrifuge (~16,000 × g). Immediately transfer the supernatant (cytoplasmic extract) to a clean pre-chilled tube the insoluble (pellet) fraction which contains nuclei treated with ice-cold 250 μl NER (nuclear extraction reagent) keep the sample on ice and continue vortexing for 15 s every 10 min, for a total of 40 min then centrifuged at maximum speed (~16,000 × g) in a microcentrifuge for 10 min. Immediately transfer the supernatant (nuclear extract) fraction to a clean pre-chilled tube. Store extracts at -80 °C until use. Cytoplasmic and nuclear protein extracts were used for Western blot analysis.

### Gene silencing with small interfering RNAs (FOXO3a)

MCF-7/MDA-MB231 cells were cultured in six-well plates until 60 % confluent and were transfected with l μg of SiRNA duplex and scrambled oligonucleotides using SiRNA transfection reagent (Sigma Aldrich sc-37887) according to the manufacturer’s instructions. 48 h after transfection, cells were treated with vernodalin or DMSO vehicle for 24 h. Then the cells were collected for Western blot analysis.

### Cell cycle analysis

Cell cycle analysis was performed using propidium iodide staining as described previously with slight modifications [37]. Briefly, after silencing with FOXO3a cells were washed in PBS and fixed in 90 % ethanol. Fixed cells were then washed twice in PBS and stained in 50 μM propidium iodide containing 5 μg/ml DNase-free RNase for 1 h, then analyzed by flow cytometry using a FACS Canto II flow cytometer (BD Biosciences, USA).

### Experimental animals and diet

The animal protocol was approved by the Institutional Animal Use and Care Committee of the University of Malaya (2014-05-07/PHAR/R/CYL). Pathogen-free female Sprague-Dawley rats (6 weeks old) were obtained from the Animal House, Faculty of Medicine, University of Malaya, Kuala Lumpur. The animals were acclimatized to standard laboratory conditions including a controlled environment at 24 ± 1 °C and 50 ± 10 % relative humidity with the alternating 12:12-h dark–light cycle for 1 week before the beginning of the study and provided with standard food pellets and tap water ad libitum.

### Cell preparation and mammary tumor induction

When the cells (LA7) were 90 % confluent, the medium was removed and the cells washed with PBS to remove dead and undetached cells. Low amount of trypsin-EDTA was added to detach cells. Cells were obtained immediately by centrifugation at 100 g for 10 min at 4 °C, washed twice with PBS and dispersed. For viability detection, the cells were stained with trypan blue and counted using a hemocytometer. Cells were eventually suspended in 300 μL of PBS. All harvested cells were used within 1 h of preparation. After one week acclimation period, rats were anesthetized using an intraperitoneal (*i.p.*) injection with a mixture of ketamine-HCl (150 mg/kg body weight) and xylazine (10 mg/kg body weight). The LA7 cells (300 μL containing 5x10^6^ cells) were inoculated subcutaneously into the mammary fat pad (left flank) of rats using a tuberculin syringe and 21G needle to initiate tumor growth. A total of twenty five female SD rats have been used in this study. The animals were divided into five groups (*n* = 5), where the Group (I) animals were kept as the normal control group (NC) and received *i.p.* injection of saline (300 μl) and served as a control. Group (II) animals were classified as LA7-induced tumor control group (TC). Groups (III) represent the low dose treatment of vernodalin in tumor bearing animals (TT+ 1 mg/kg-Vernodalin), Groups (IV) represent the high dose treatment of vernodalin in tumor bearing animals (TT+ 10 mg/kg-Vernodalin), and Group (V) represent the Tamoxifen treatment in tumor bearing animals (TT+ 1 mg/kg Tamoxifen). For Group III to V the Vernodalin and Tamoxifen (from Sigma Aldrich, USA) were dissolved in saline separately and the treatment was performed by intraperitoneal (*i.p.*) injection three times/week after the appearance of tumor (approximate size is 2 cm in 2 weeks) for another three weeks. The body weight of each rat was recorded weekly. At the end of the experimental period (After five weeks), rats were fasted overnight and the body weight of each rat was monitored. Then, the rats were anaesthetized and sacrificed by decapitation. Mammary tissue samples were obtained washed twice with ice-cold 0.1 M phosphate buffered saline (PBS), weighed and fixed in 10 % neutral-buffered formalin for immunohistochemical analyses.

### Tumor growth and measurement

The latency period and tumor incidence was determined as tumor growth landmarks [38]. All animals were monitored for mammary tumor development. Tumor diameters and animal weight were measured every week. The tumor mass was measured horizontally and vertically using a digital caliper. Volume of tumor (V) was calculated by the formula determined by Carlsson: V = (ab2)/2, where ‘a’ and ‘b’ is the longest and shortest diameters of the tumor, respectively [39].

### Histopathological examination

Kidney and liver tissues were fixed in 10 % buffered formalin, embedded in paraffin using a conventional automated system. The blocks were cut to obtain 5 mm thick sections and stained with hemotoxylin–eosin. Serial paraffin sections of each tissue image were captured by light microscopy (Nikon XDS-1B).

### Immunohistochemical analysis

Paraffin embedded tissue sections of 5 μm thickness were rehydrated first in xylene and then in graded ethanol solutions. Then, the slides were incubated with the HistoVT (10x, pH 7.0) (Nacali Tesque, Tokyo, Japan) antigen retrieval solution for 20 min in 90 °C followed by cooling in room temperature. Next, the slides were blocked with 5 % BSA in Tris-buffered saline-Tween 20 (TBS-T) for 2 h before the sections were immunostained with primary antibodies of PCNA, Ki67, FOXO3a, p-FOXO3a and p27Kip1 (Cell Signaling Technology, USA) diluted 1:100 with 5 % BSA in TBS-T and incubated overnight at 4 °C. After washing the slides thrice with TBS-T, the sections were incubated with respective secondary antibodies with 5 % BSA in TBS-T and incubated for 2 h at room temperature. Sections were then washed with TBST and incubated for 5-10 min in a Peroxidase stain DAB kit as per the instructions provided by the manufacturer (Dako, Glostrup, Denmark). Counter staining was performed using hematoxylin (Cell Path, UK), and the slides were photographed in light microscope (BX51, Olympus, Japan).

### Statistical analysis

Statistical analyses were processed according to conventional procedures using the Statistical Program of Social Sciences (SPSS) software for Windows, Version 12.0 (Post-hoc, Tukey’s test). A *P* value <0.05 was considered statistically significant.

## Results

### Effect of vernodalin treatment on FOXO3 and its downstream target molecules

**Fig. 1:**
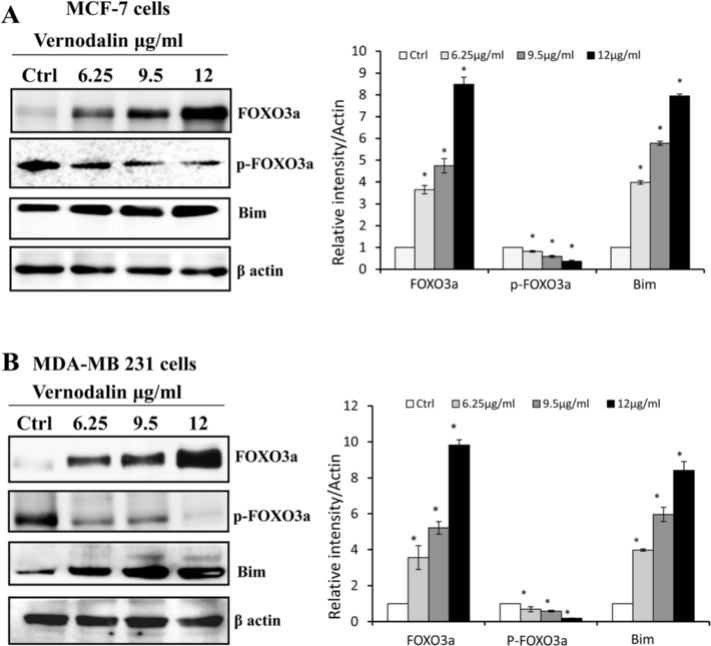
Effect of Vernodalin on modulation of FOXO3a and Bim. Expression of FOXO3a/p-FOXO3a (Ser253) and Bim in breast cancer cells of MCF-7 (**a**) and MDA-MB231 (**b**). Whole cell lysates were prepared from the breast cancer cell lines after the vernodalin treatment for 24 h. The expression of FOXO3a, phosphorylated FOXO3a, and Bim were analysed by Western blots as described in “Materials and Methods”. β-actin was used as loading control. The data are representative of three experiments. Respective blots were quantified using ImageJ software. Data’s are expressed as mean ± S.D. All the treatment groups were compared with control. “*” denotes statistically significant at *P* < 0.05

**Fig. 2:**
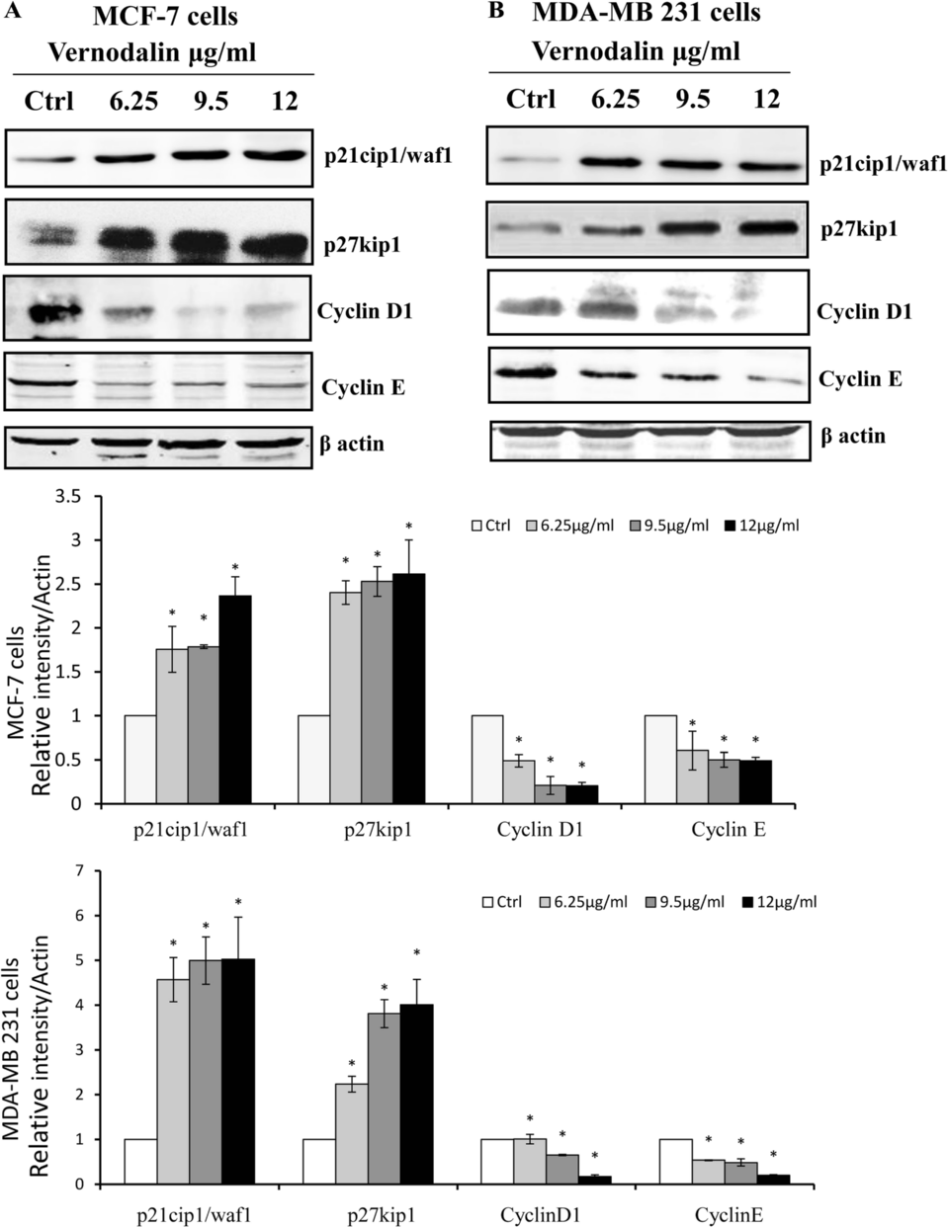
Effect of Vernodalin on modulation of cell cycle proteins. **a** & **b** Shows the Western blots expression of p27kip1, p21cip1/waf1, cyclin D1 and cyclin E in breast cancer cells of MCF-7 and MDA-MB231. β-actin was used as loading control. The data are representative of three experiments. Respective blots were quantified using ImageJ software. Data’s are expressed as mean ± S.D. All the treatment groups were compared with control. “*” denotes statistically significant at *P* < 0.05

Several studies showed that phytochemicals such as genistein exert anti-cancer effect via activation of FOXO3a signalling [40, 41]. Therefore, in this study we embarked to evaluate whether vernodalin (a cytotoxic compound isolated from *Centratherum anthelminticum* seeds) could affect FOXO3a expression in human breast cancer cell-lines (MCF-7 and MDA-MB231). Our results showed that vernodalin dose dependently (6.25, 9.5 and 12 μg/ml) induced higher expression of FOXO3a in both MCF-7 and MDA-MB231 cells. In addition immunoblotting analysis showed a reduction of the phosphorylated forms of FOXO3a at the critical phosphorylation sites (Ser253) after vernodalin treatment (6.25, 9.5 and 12 μg/ml) as compared with untreated control cells (Fig. 1a and b). The cell cycle progression is regulated by cyclin/cyclin-dependent kinase (CDK) complexes, as the uncontrolled expressions of cyclins and/or CDKs may lead to cell cycle dysregulation and tumorigenesis. FOXO3a controls cell cycle progression by regulating the downstream expression level of cell cycle inhibitor p27Kip1 and p21 (Cip1/waf1). Results showed that p27Kip1 and p21Cip1/waf1 were up-regulated, whereas the levels of cyclin D1 and cyclin E were decreased in response to vernodalin treatment (Fig. 2a and b). Similarly, the pro-apoptotic protein Bim, another downstream target of FOXO3a was increased in both breast cancer-lines cells at 24 h after vernodalin treatment (6.25, 9.5 and 12 μg/ml) as compared with untreated control cells Fig. 1a and b.

### FOXO3a subcellular localization is affected by vernodalin treatment

**Fig. 3:**
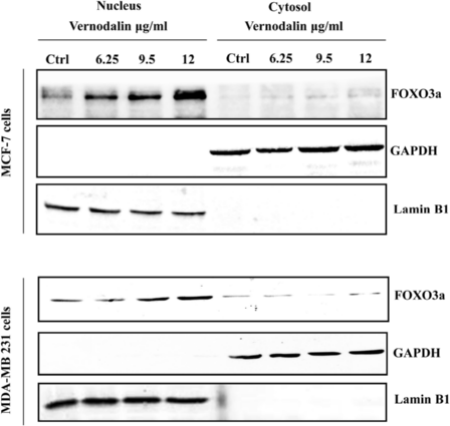
FOXO3a cytoplasmic and nuclear protein expression in MCF-7 and MDA-MB231 cells after vernodalin treatment. MCF-7 and MDA-MB231 cells were treated with vernodalin for the indicated time periods, and the subcellular fractions were then isolated and immunoblotted for FOXO3a. Lamin B1 and GAPDH were used as markers of the nucleus and cytoplasm respectively. Vernodalin treatment caused the translocation of FOXO3a from the cytoplasm to the nucleus. Results are representative of three independent experiments

Previously, we showed that FOXO3a is upregulated in vernodalin treated breast cancer cells. Next, we seek to investigate if vernodalin treatment could relocate FOXO3a protein to the nucleus in order to exert its function. We treated MCF-7 and MDA-MB231 cells with vernodalin for 24 h and the intracellular localization of FOXO3a was analysed by immunofluorescent staining. As shown in Additional file 2: Figure S2 and Additional file 3: Figure S3, FOXO3a resided in the cytoplasm, with negligible nuclear staining in control untreated cells. Interestingly, vernodalin treatment induced a change in the subcellular localization of FOXO3a, with a significant proportion of the protein being translocated to the nucleus after 24 h treatment, comparable to standard drug Doxorubicin (Additional file 2: Figure S2 and Additional file 3: Figure S3). To confirm this, we performed nuclear/cytoplasmic fractionation of breast cancer cells treated with vernodalin. Western blot results showed higher expression of FOXO3a in the nucleus compared with the cytoplasmic fractionation after vernodalin treatment in MCF-7 and MDA-MB231 cells (Fig. 3). This correlates with nuclear translocation of the FOXO3a transcription factor that may contribute to FOXO3a activation and induction of cell death during vernodalin treatment.

### FOXO3a silencing by siRNA abrogates vernodalin-induced cell death

**Fig. 4:**
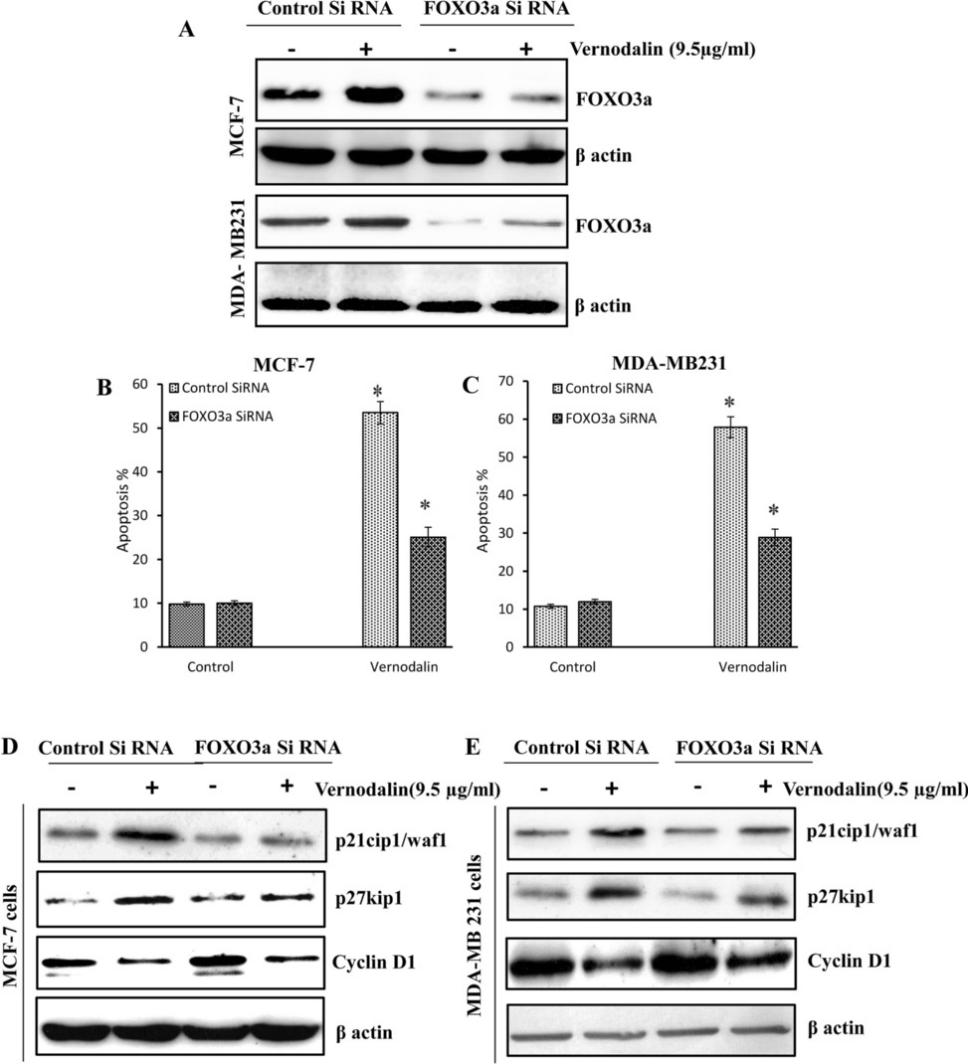
Effect of FOXO3a silencing during vernodalin treatment in MCF-7 and MDA-MB231 cells. **a** Cells were pre-treated with scrambled siRNA or siFOXO3a for 48 h, followed by vernodalin (9.5 μg/ml) treatment for 24 h in MCF-7 and MDA-MB231 cells. All groups of cells were analysed by using Western blots. **b** & **c** Cells were fixed in ethanol and stained with propidium iodide, and then the percentage of apoptotic cells was determined by flow cytometry. Data are presented as means ± SD of three independent experiments (*P < 0.05). **d** & **e** Cells were pre-treated with scrambled siRNA or siFOXO3a for 48 h, followed by vernodalin (9.5 μg/ml) treatment for 24 h in MCF-7 and MDA-MB231 cells. Protein lysates were prepared at the times indicated and the levels of p27kip1, p21cip1/waf1 and cyclin D1 were analysed by using Western blots. Data are presented as means ± SD of three independent experiments

In order to confirm the relevance of FOXO3a in vernodalin mediated apoptosis, we performed gene silencing experiments by transfecting MCF-7 and MDA-MB231 cells with siRNA specific for FOXO3a. As shown in Fig. 4a, vernodalin treated or control siRNA transfected cells have higher FOXO3a protein level. In contrast, FOXO3a silencing by siRNA abrogated vernodalin-induced expression of FOXO3a protein as determined by Western blot. In addition, cell cycle analysis showed that silencing of FOXO3a by siRNA can partially rescue cells from vernodalin-mediated cell apoptosis. This is evident from the decrease in the proportion of cells in the sub-G1 phase in siFOXO3a-transfected breast cancer cells when compared with the control siRNA-transfected cells (Fig. 4b and c). To further show that FOXO3a has an essential role in response to vernodalin treatment, we transfected the MCF-7 and MDA-MB231carcinoma cells with either a FOXO3a-specific siRNA or control siRNA and studied the levels of cell cycle regulatory proteins after vernodalin treatment. Western blots analysis showed that the FOXO3a-specific siRNA, but not control siRNA, inhibited the induction of p27Kip1, p21cip1/waf1, and increased the cyclin D1 protein levels (Fig. 4d and e). These results suggested that vernodalin targets FOXO3a to mediate apoptosis in breast cancer cells.

### Vernodalin-induced activation of FOXO3a is dependent on PI3K/Akt signaling pathway

**Fig. 5:**
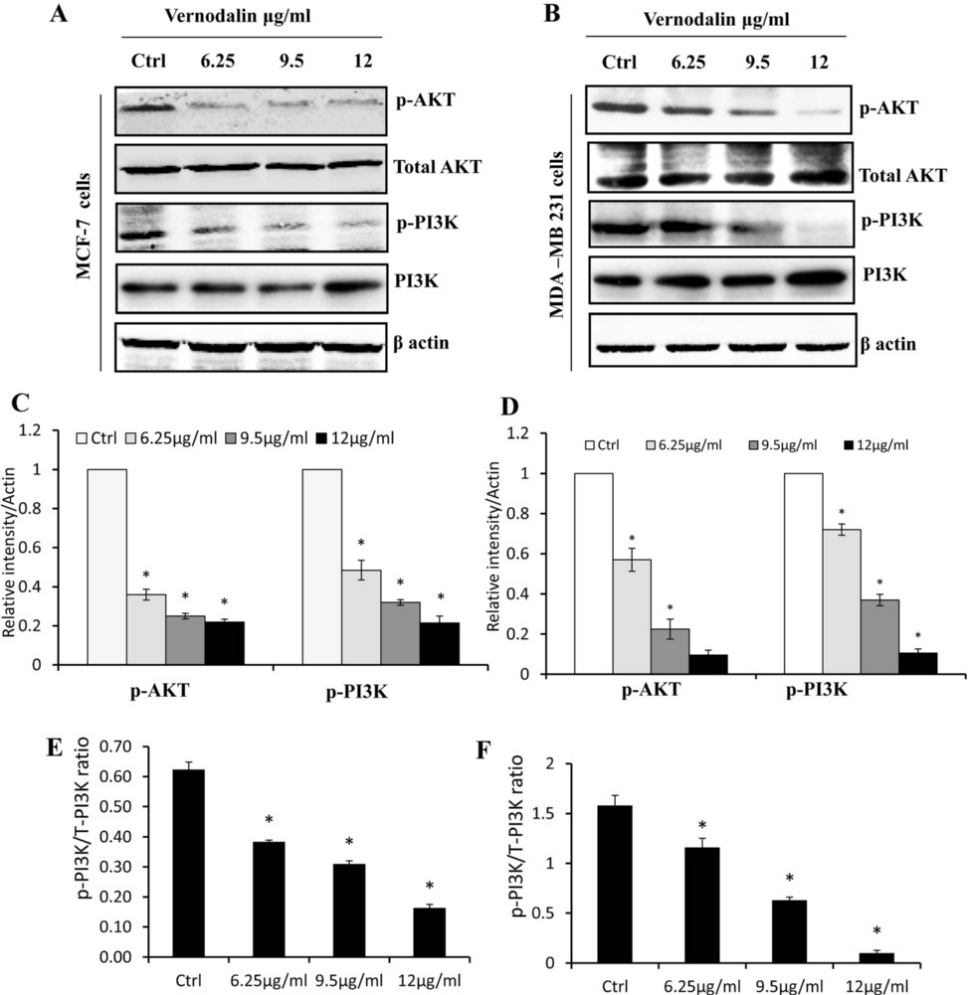
Effect of vernodalin on modulation of PI3K/Akt signalling. MCF-7 cells and MDA-MB231 were left untreated or treated with vernodalin at indicated concentration for 24 h (**a** & **b**) Expression of Total Akt, p-Akt, Total PI3K and p-PI3K were analysed by immunoblot analysis. Respective blots were quantified using Image J software (**c** & **d**). E & F shows the ratio between P-PI3K and total PI3K protein. All the treatment groups were compared with control. “*” denotes statistically significant at *P* < 0.05. Results are shown as percentage values ± SD of three independent experiments

In our study, we examined the role of vernodalin on PI3K/Akt signaling in both breast cancer cell-lines. In control cells, Akt and PI3K are predominantly activated, while vernodalin treatment markedly reduced the phosphorylation of Akt and PI3K as compared with control (Fig. 5a and b). This correlates with our previous Western blot data, which showed vernodalin treatment, lead to decrease level of p-FOXO3a. The Akt kinase activity was further assessed by the phosphorylation of GSK-3 protein through immunoprecipitation and Western blots. Immunocomplex kinase assay of Akt revealed that treatment with vernodalin for 24 h significantly abolished Akt kinase activity in MCF-7 and MDA-MB231 cells (Additional file 4: Figure S4). We have also checked whether any feedback regulation during FOXO3a silencing and analysed the protein levels of Akt and p-Akt during vernodalin treatment in control, FOXO3a specific siRNA and we could not find the significant changes in regarding with the feedback regulation (Additional file 5: Figure S5). Taken together, these observations suggest the importance of Akt signaling cascade in the activation of transcription factor FOXO3a and the extent of cell death caused by vernodalin in MCF-7 and MDA-MB231 cells.

### Tumor development, body weight, tumor size and volume

During the experimental period, the rats tolerated the subcutaneous administration of LA7 rat mammary tumor cells and vernodalin treatment by intraperitoneal injection. There were no clinical signs of toxicity related death in experimental groups. Histology of the liver and kidney tissues after vernodalin treatment (1 mg/kg and 10 mg/kg) was shown in the Additional file 6: Figure S6. As hematoxylin–eosin (H–E) staining these organs did not revealed histopathological abnormalities, degeneration, lesions or necrosis during the treatment. Liver sections of control and vernodalin treated rats exhibiting a concentric arrangement of the hepatocytes with sinusoidal cards around the central vein and portal tracts. Kidney sections of control and vernodalin treated rats showing normal renal tubules, corticomedullary junction, and glomeruli within the cortex region. The breast tumors started to develop within 7-10 days after LA7 cells injection. The body weight, tumor volume and the tumor percentage (%) of control and treated animals is represented in Table 1. There was no significant body weight decreases in the tumor bearing rats (Group II-V) when it was compared with control rats (Group I). The tumor volume in untreated LA7 breast tumor bearing rats was enlarged up to 2143 ± 363 mm^3^, whereas a significant decrease in tumor volume (1182 ± 352 mm^3^, 524 ± 261 mm^3^) was observed in the treatment groups (low and high dose of vernodalin), comparable to the standard drug Tamoxifen-treated group 562 ± 145 mm^3^ (Table 1).

**Table 1:** Treatment effect of vernodalin and TAM on animal body weight (g), tumor volume (mm^3^) and tumor reduction percentage (%) in LA-7-induced breast cancer rats

Group	Treatment groups	Body weight (g)	Tumor volume (mm^3^)	Tumor reduction percentage (%)
I	NC	247.3 ± 1.5	0	0 %
II	TC	213.13 ± 1.32	2143 ± 363	0 %
III	TT (1 mg/kg vernodalin)	219.14 ± 7.2	1182 ± 352*	44.84 %
IV	TT (10 mg/kg vernodalin)	221.57 ± 5	524 ± 261*	75.6 %
V	TT (1 mg/kg TAM)	224.88 ± 5.7	562 ± 145*	73.78 %

Each value represents mean ± S.D. of given number of animals (*n* = 5). “*” denotes statistically significant at *p* < 0.05 compared to control group

### Vernodalin inhibited rat breast tumor growth in vivo by targeting FOXO3a

**Fig. 6:**
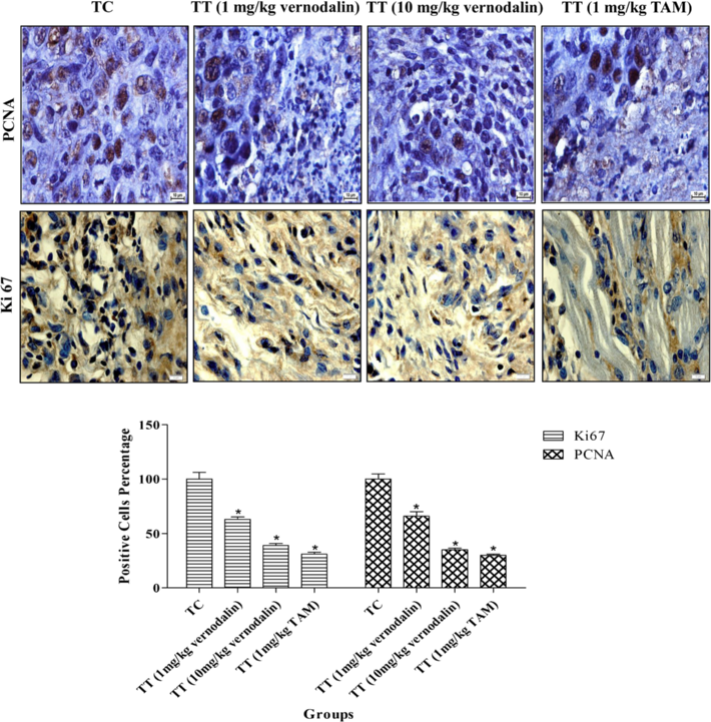
Effect of vernodalin treatment on proliferation markers (Ki-67 and PCNA) in breast tumor tissue. Sprague dawley rats were implanted with LA7 cells. Rats were treated with low and high dose of vernodalin (1 or 10 mg/kg) and the standard drug Tamoxifen (1 mg/kg). Tumor tissues were collected in 10 % formalin and blocks were prepared in paraffin and immunohistochemistry of PCNA and Ki67 were performed as described in “Materials and Methods”. Photomicrographs show representative pictures from five independent tumor samples. Bar =10 μm. Quantification of Ki-67 and PCNA positive cells in tumor tissues was shown (*P < 0.05)

**Fig. 7:**
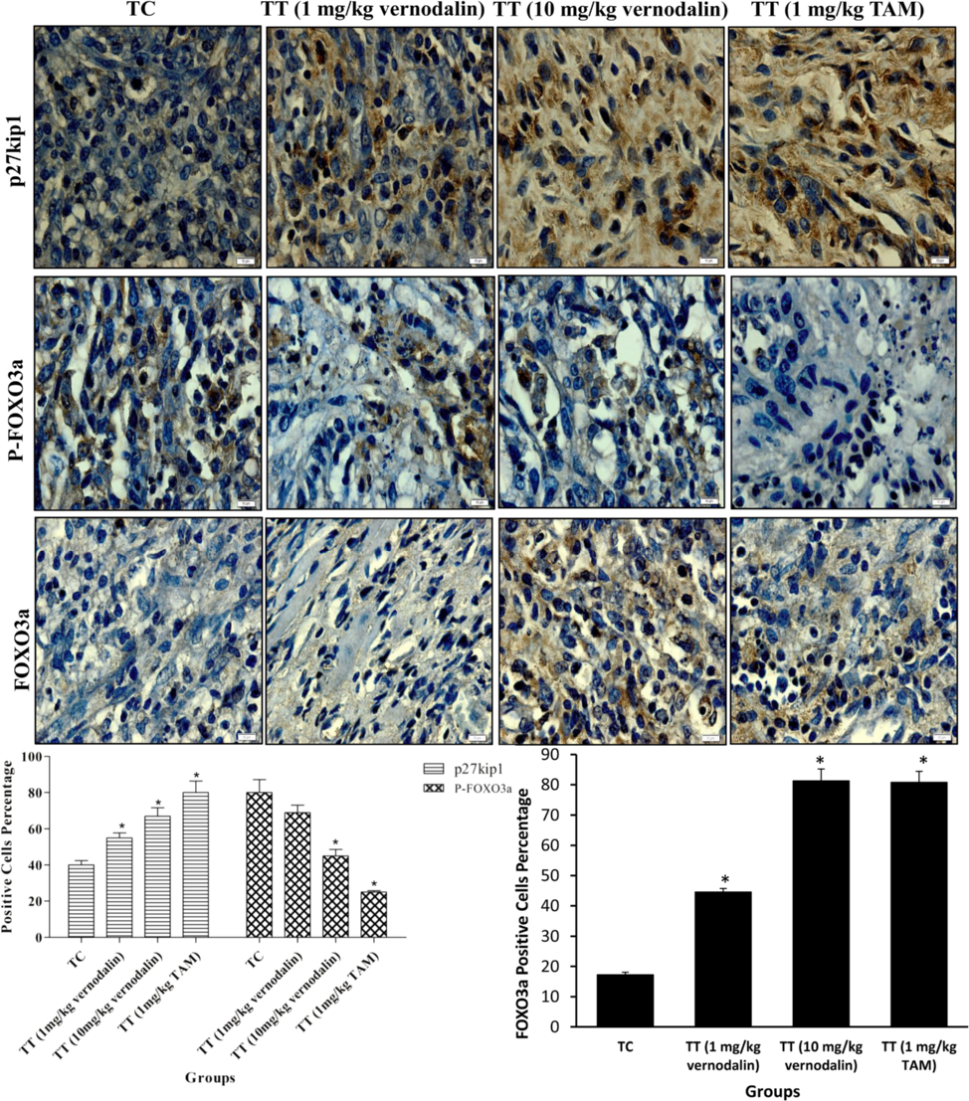
Effect of vernodalin on expression of cell cycle inhibitor p27/Kip1, p-FOXO3a and FOXO3a in breast tumor tissue. Sprague dawley rats were implanted with LA7 cells. Rats were treated with low and high dose of vernodalin (1 or 10 mg/kg) and the standard drug Tamoxifen (1 mg/kg). Tumor tissues were collected in 10 % formalin and blocks were prepared in paraffin and immunohistochemistry of p27kip1, p-FOXO3a and FOXO3a were performed as described in “Materials and Methods”. Photomicrographs show representative pictures from five independent tumor samples. Bar =10 μm. Quantification of p27/Kip1, p-FOXO3a and

To assess the proliferation rate of breast tumor cells, the proliferation indexes were evaluated by Ki-67 and PCNA staining. The untreated LA7 breast tumor bearing rats (Group II) revealed intensive staining of PCNA and Ki-67 (Fig. 6), whereas, the vernodalin treated animals (Group III and IV; low and high dose respectively) exhibited a substantial decrease in the numbers and intensity of cell proliferation markers such as Ki67 and PCNA, comparable to a standard drug, Tamoxifen (Group V). As we showed that vernodalin could inhibit phosphorylation of FOXO3a in vitro, next we examined the phosphorylation status of FOXO3a and total FOXO3a protein in tumor tissues by immunohistochemistry. Treatment of rats with vernodalin inhibited the phosphorylation of FOXO3a and increases the total FOXO3a protein levels as compared to untreated control (Fig. 7). Since cell cycle inhibitor p27Kip1 is one of the targets of the FOXO transcription factor, we next examined the effect of vernodalin on the expression of p27Kip1 (Fig. 7). Immunohistochemistry result reveals that p27Kip1 expression is higher in rats treated with low and high dose vernodalin (Group III and IV) compared with untreated LA7 tumor bearing animals (Group II). These data suggest that vernodalin can cause growth arrest in tumor cells in vivo by inducing the expression of FOXO3a and p27Kip1.

## Discussion

Dysregulated proliferation is one of the main mechanisms for breast tumorigenesis, which involves different stages such as initiation, promotion and progression of tumor growth, thereby increasing the tumor burden and the increase the chances of metastasis. This dysregulation mainly occurs due to disrupted G1/S phase cell cycle transition during malignant neoplasm development [42]. Recently, research on the therapeutic activities of natural products in cancer therapy with diverse roles and targets are gaining attention [43–45]. Our previous studies revealed that vernodalin isolated from *C. anthelminticum* seeds possess anticancer property in breast and skin cancer model [37, 46]. However, the precise mechanism whereby vernodalin influences apoptosis in breast cancer remains unclear. FOXO transcription factors play an important role in tumor suppression by upregulation of pro-apoptotic genes, such as Bim and p27Kip1 in various tumors [47–50]. In this study, we evaluated the expression of FOXO3a and other important cell cycle molecules in response to vernodalin treatment. Subsequently, we examined the effect of vernodalin on PI3K/Akt pathway and provided evidences showing that FOXO3a activation could inhibit breast cancer cell proliferation and tumorigenesis.

Cell-cycle dysregulation is an attribute of tumor cells, thus cell-cycle arrest has become the major focus of anti-cancer effects. Anti-tumor agents can cause cell cycle arrest in various phases by regulating the cell-cycle machinery [51]. We previously reported that vernodalin induced cell cycle arrest at G0/G1 stage in breast cancer cells [37]. In the present study, we observed loss of cyclin D1, cyclin E expression and upregulation of p21 and p27 levels during vernodalin treatment. The p21 and p27 belong to the Cip/Kip family negatively regulate the cell cycle progression through inhibition of CDK-cyclin complexes [52]. On the other hand, cyclin D1 and cyclin E are major cell cycle regulatory proteins and mainly involved in the G1/S phase transition in normal cells. Over expression of these cyclin altered the cell cycle progression and closely associated with malignancy. The dysregulated cell proliferation plays an important role in multistage oncogenesis, which could caused by over expression of cyclins D1, E, and CDK4 complexes [53, 54]. The G1/S-phase transition is tightly regulated by CDKIs such as p21 and p27 via inactivation of G1 (CDK) complexes [55, 56]. Taken together, our data reveal a novel cell cycle regulating property of vernodalin via G1-phase arrest.

Apoptotic cell death is a complex program mainly controlled by the Bcl-2 family proteins. We previously reported that the apoptosis induction in vernodalin-treated MCF-7 and MDA-MB231 cells is associated with upregulation of Bax (a Bcl-2 family member), cytochrome c release and caspases activation [37]. However, it was known that the presence of BH3-only molecules like Bim is required for direct activation of Bax at the mitochondria. In this study, we observed an increase in the protein expression of Bim after vernodalin treatment in breast cancer cells (Fig. 1a and b). Bim was also shown to be a direct downstream target of FOXO3a [57]. It is widely known for its pro-apoptotic functions in mitochondria and it can induce apoptosis by interacting with proteins such as Bcl-xL and Bcl-2 that shows anti-apoptotic function [58]. PI3K/Akt pathway is a cell survival pathway that is important for normal cell growth and proliferation. Numerous researches in breast cancer have shown that deregulation of this pathway is implicated in tumorigenesis and hence this has become an important target for breast cancer treatment. Notably, this pathway regulates the development and progression of human breast cancer by modulating the status of anti-apoptotic proteins, by mediating cell cycle arrest and inhibiting aerobic glycolysis via Akt which would preferentially kill tumor cells via oxidative stress [56, 59]. As the amplification of PI3K/Akt signal transduction is the main force that drives cellular growth, thus abrogation of PI3K or Akt function might be crucial for cancer therapy. Of note, phosphorylated Akt is an attractive molecular target because it contributes to the development of breast cancer and confers resistance to conventional therapies [60] In this study, we observed decreased expression of p-Akt and p-PI3K after vernodalin treatment in both breast cancer cell-lines (Fig. 5) and this was further confirmed by in vitro kinase assay of Akt showed diminished phosphorylation of fusion protein GSK in vernodalin treated breast cancer cells and reflects the inhibitory effect of vernodalin on Akt kinase which clearly demonstrated that nuclear localization of FOXO3a was dependent on Akt signalling [61]. GSK3β is reported to regulate cell cycle analysis and apoptosis, Akt prevents the apoptotic activity of GSK3β by phosphorylation [62]. Our results suggested that vernodalin reflects the decrease in the Akt kinase activity as evaluated by the phosphorylation of GSK-3β protein as shown in (Additional file 4: Figure S4).

FOXO3a is an important downstream effector of the PI3K/Akt pathway. These proteins have emerged as major regulators that control cellular activities, through the orchestration of different patterns of gene expression in response to diverse stimuli [63]. Activation of Akt leads to phosphorylation of FOXO3a, resulting in its deactivation through nuclear export, while inhibition of Akt leads to dephosphorylation, nuclear localization and activation of FOXO3a [64]. The cytosolic retention of FOXO3a prevents the transactivation of downstream target genes such as Bim and p27Kip1. We hypothesized that the inhibition of Akt phosphorylation by vernodalin would lead to nuclear accumulation of FOXO3a and increased transcription of downstream responsive genes. Studies have shown that FOXO3a is dephosphorylated and activated by LY294002 (a potent inhibitor of PI3K), which correlates with upregulation of p27Kip1 [65]. It has been previously demonstrated that FOXO3a pathway can induce Bim expression and subsequently causing cell death in cancer models, such as MCF-7, mice xenografts model of pancreas tumor and lymphoma cells [66–68]. In agreement with the hypothesis, our study provides clear evidence that vernodalin treatment resulted in the activation (Fig. 1) and nuclear translocation (Fig. 3, Additional file 2: Figure S2 & Additional file 3: Figure S3) of FOXO3a in breast cancer cells via PI3K/Akt inhibition (decrease phosphorylation status of PI3K/Akt and increase translocation of FOXO3a to the nucleus). Furthermore, silencing of the endogenous FOXO3a expression by siRNA rescued the breast carcinoma cells from undergoing proliferative arrest and apoptosis in response to vernodalin treatment, suggesting that the induction of FOXO3a expression has a direct role in mediating the effect of vernodalin. Transiently silenced FOXO3a also reversed the down-regulation of p21Cip1 and p27Kip1 cell cycle inhibitory proteins and increased cyclinD1 during vernodalin treatment in MCF-7 and MDA-MB 231cells (Fig. 4). These findings indicate that silencing of FOXO3a and vernodalin treatment in breast cancer cells modulates the expression of cyclinD1, p21Cip and p27Kip1 is FOXO3a/PI3K/Akt dependent manner. These results are consistent with previous published data showing that lapatinib also targets FOXO3a in breast cancer cells to induce apoptosis [69]

**Fig. 8:**
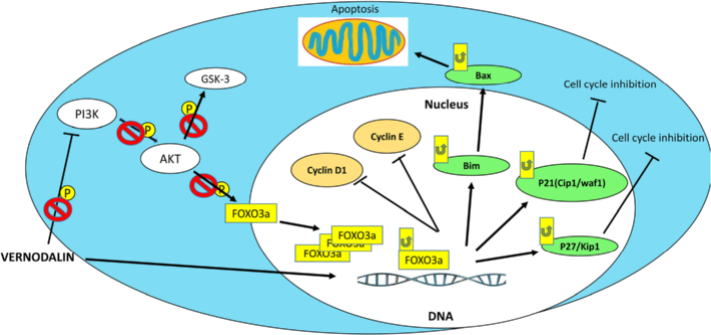
Schematic representation of mechanisms underlying vernodalin-induced cytotoxicity in breast cancer cells. Vernodalin treatment inhibits the activation of PI3K/Akt pathway, which enhances accumulation of nuclear FOXO3a in MCF-7 and MDA-MB231 cells. Nuclear FOXO3a promotes the transcription and expression of Bim, which could activate Bax and causes cell death or apoptosis. On the other hand, FOXO3a upregulates p21Cip1/waf1, p27Kip1, downregulates cyclin D1 and cyclin E leading to cell cycle arrest

For in vivo study, we used rat mammary tumor cells (LA7) to induce mammary tumors in the right flank of rats to produce malignancy. Using this method, the mammary gland tumor developed within 7-10 days at the site of injection [70]. Current findings indicate that the applied low (1 mg/kg) or high (10 mg/kg) vernodalin dosages did not produce any toxicity sign in rats. Using these dosages, we observed a significant decrease in tumor volume in treatment groups, indicating vernodalin could suppress breast tumor growth in vivo. The result was comparable to a standard drug tamoxifen, which has been widely used for the treatment of breast cancer [71]. The anti-proliferative action of vernodalin was further confirmed using cell proliferative markers, namely PCNA and Ki67. PCNA (cell cycle related protein) is synthesized in early G1 to S-phase function in the cell cycle progression; DNA replication and repair [72]. Whereas Ki67 (nuclear protein) is required for maintaining cell proliferation and commonly used to evaluate the solid tumor cell proliferation [73]. In the current study, we observed increased expression of PCNA and Ki67 in breast tissue of control/untreated group. Concomitantly, these proliferative markers expression was efficiently decreased after vernodalin treatment (Fig. 6), indicating the inhibition of mammary gland tumorigenesis. Moreover, vernodalin induced the expression of FOXO3a protein and their target genes p27Kip1 in breast tumor tissues and decrease the level of p-FOXO3a protein, consistent with our in vitro studies (Fig. 7). These findings prove that vernodalin can inhibit the cell proliferation and breast carcinogenesis through G1/S phase cell cycle arrest. A schematic representation of vernodalin mechanism is shown in Fig. 8.

## Conclusion

It is obvious that vernodalin interferes with cellular processes by targeting different intracellular molecules. Using in vitro breast cancer cell-line model, we demonstrated that the cytotoxicity of vernodalin is mediated through FOXO3a activation. In addition, in vivo experiments reveal that vernodalin inhibits rat breast tumor growth by targeting FOXO3a and its downstream molecule, p27Kip1. Our results suggest that a combination of PI3K/Akt/FOXO signaling pathway is responsible for the cytotoxicity of vernodalin against breast cancer cells and this could also serve as a new target gene for the therapeutic or preventive intervention in breast cancer as well as other cancer typologies.

## Additional files

Additional file 1: Figure S1. Chemical structure of Vernodalin. (JPG 130 kb) Additional file 2: Figure S2. Vernodalin treatment induces FOXO3a nuclear translocation in MCF-7 cells. MCF-7 cells were treated with DMSO (control) or indicated concentration of vernodalin for 24 h. Immunofluorescent staining was then performed using the FOXO3a antibody (green) and stained with Hoechst 33258 (blue). Images were acquired using cellomic HCS array scan reader (objective 20X). Representative figures (control, 6.25, 9.5 μg/ml of vernodalin and Doxorubicin 3 μg/ml) were shown. Bar chart shows average fluorescence intensities of FOXO3a accumulation in the nucleus. Data were mean ± SD of fluorescence intensity readings representative of three independent experiments. (**P* < 0.05). (JPG 536 kb) Additional file 3: Figure S3. Vernodalin treatment induces FOXO3a nuclear translocation in MDA-MB-231 cells. MDA-MB231 cells were treated with DMSO (control) or indicated concentration of vernodalin for 24 h. Immunofluorescent staining was then performed using the FOXO3a antibody (green) and stained with Hoechst 33258 (blue). Images were acquired using Cellomic HCS array scan reader (objective 20X). Representative figures (control, 6.25, 9.5 μg/ml of vernodalin and Doxorubicin 3 μg/ml) were shown. Bar chart shows average fluorescence intensities of FOXO3a accumulation in the nucleus. Data were mean ± SD of fluorescence intensity readings representative of three independent experiments. (**P* < 0.05). (JPG 573 kb) Additional file 4: Figure S4. AKT kinase assay. Akt enzymatic activity was evaluated by phosphorylation of a GSK-3 fusion protein after immunoprecipitation of Akt using a nonradioactive Akt kinase kit as described in the Materials and Methods section. The relative intensity of the p-GSK3α/β band reflected the AKT kinase activity and Total-AKT was immunoblotted as controls. GSK-3 phosphorylation bands were analysed densitometrically. All the treatment groups were compared with control. “*” denotes statistically significant at *P* < 0.05. Results are shown as percentage values ± SD of three independent experiments. (JPG 318 kb) Additional file 5: Figure S5. Effect of FOXO3a-specific siRNA on expression of AKT and p-AKT in response to vernodalin treatment in MCF-7 and MDA-MB231 cells. Cells were pre-treated with scrambled siRNA or siFOXO3a for 48 h, followed by vernodalin (9.5 μg/ml) treatment for 24 h in MCF-7 and MDA-MB231 cells. Protein lysates were prepared at the times indicated and the levels of AKT and p-AKT were analysed by using Western blots. Data are presented as means ± SD of three independent experiments. (JPG 255 kb) Additional file 6: Figure S6. Histopathological study of kidney and liver tissues from control or vernodalin treated rats. A.control (untreated). B. vernodalin 1 mg/kg- treated. C. vernodalin 10 mg/kg- treated. (n = 5). Liver sections of control and vernodalin treated rats showing normal architecture of hepatocytes with visible central vein and normal arrangement of hepatocytes. Kidney sections of control and vernodalin treated rats showing normal glomerular, tubular sections. Representative micrographs of histologic sections were shown at 20× magnification. (JPG 396 kb)
