# Supplementary figures and images for: Forkhead Box Transcription Factor (FOXO3a) mediates the cytotoxic effect of vernodalin in vitro and inhibits the breast tumor growth in vivo
Source: J Exp Clin Cancer Res. 2015 Dec 8;34:147. doi: 10.1186/s13046-015-0266-y (PMC4672543; doi:10.1186/s13046-015-0266-y)

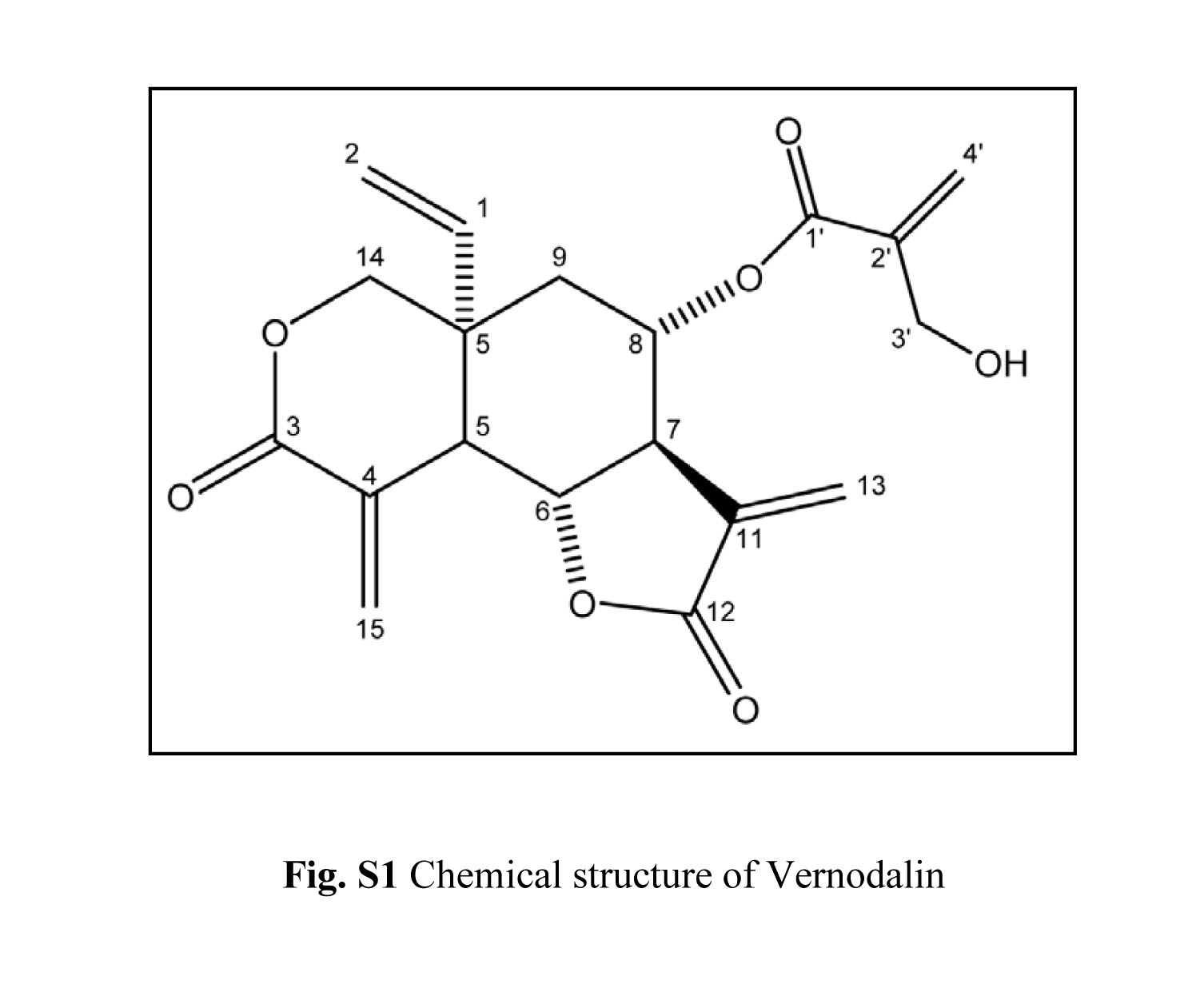

Supplement: Additional file 1: Figure S1. — Chemical structure of Vernodalin. (JPG 130 kb) [file 13046_2015_266_MOESM1_ESM.jpg]

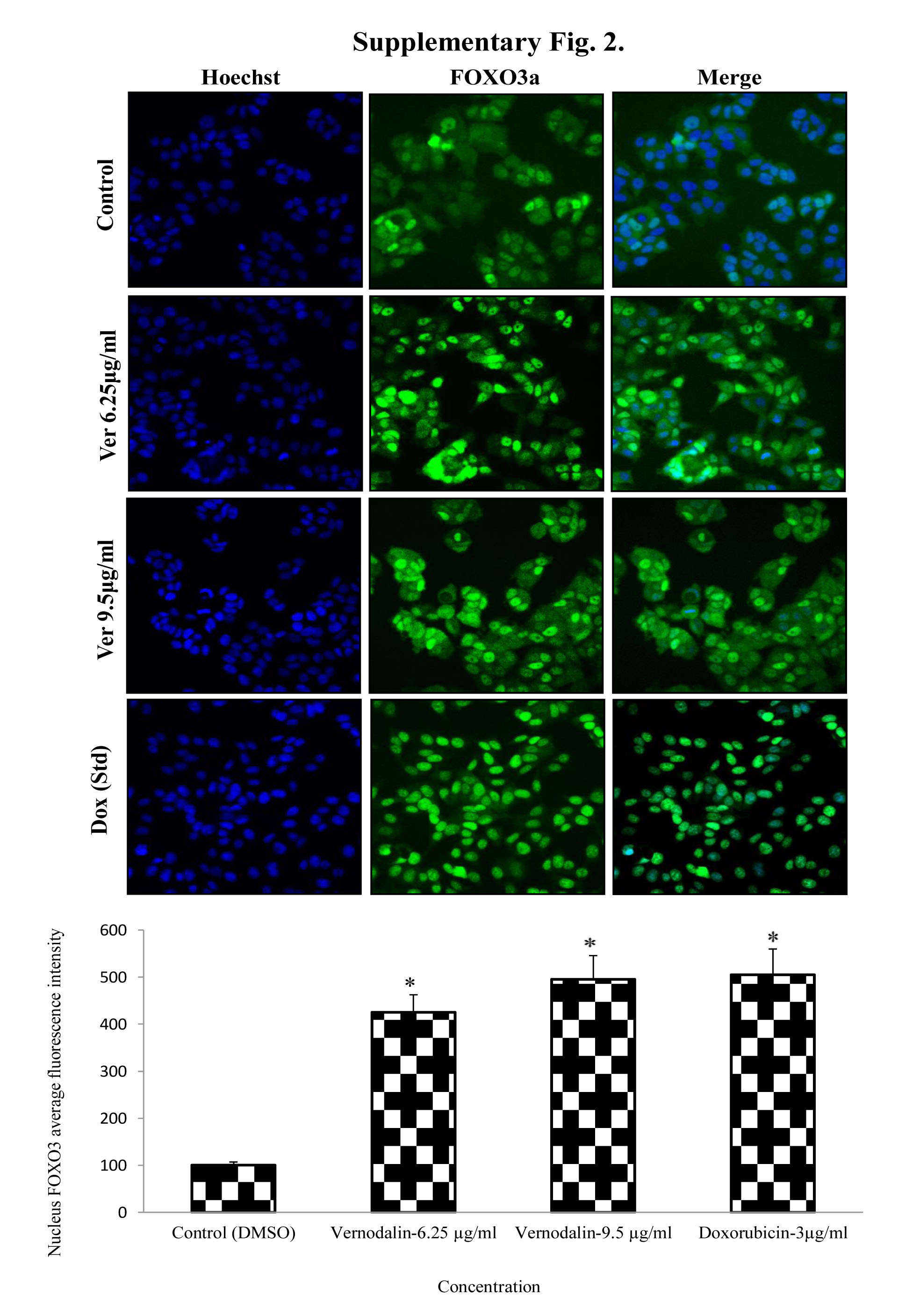

Supplement: Additional file 2: Figure S2. — Vernodalin treatment induces FOXO3a nuclear translocation in MCF-7 cells. MCF-7 cells were treated with DMSO (control) or indicated concentration of vernodalin for 24 h. Immunofluorescent staining was then performed using the FOXO3a antibody (green) and stained with Hoechst 33258 (blue). Images were acquired using cellomic HCS array scan reader (objective 20X). Representative figures (control, 6.25, 9.5 μg/ml of vernodalin and Doxorubicin 3 μg/ml) were shown. Bar chart shows average fluorescence intensities of FOXO3a accumulation in the nucleus. Data were mean ± SD of fluorescence intensity readings representative of three independent experiments. (*P < 0.05). (JPG 536 kb) [file 13046_2015_266_MOESM2_ESM.jpg]

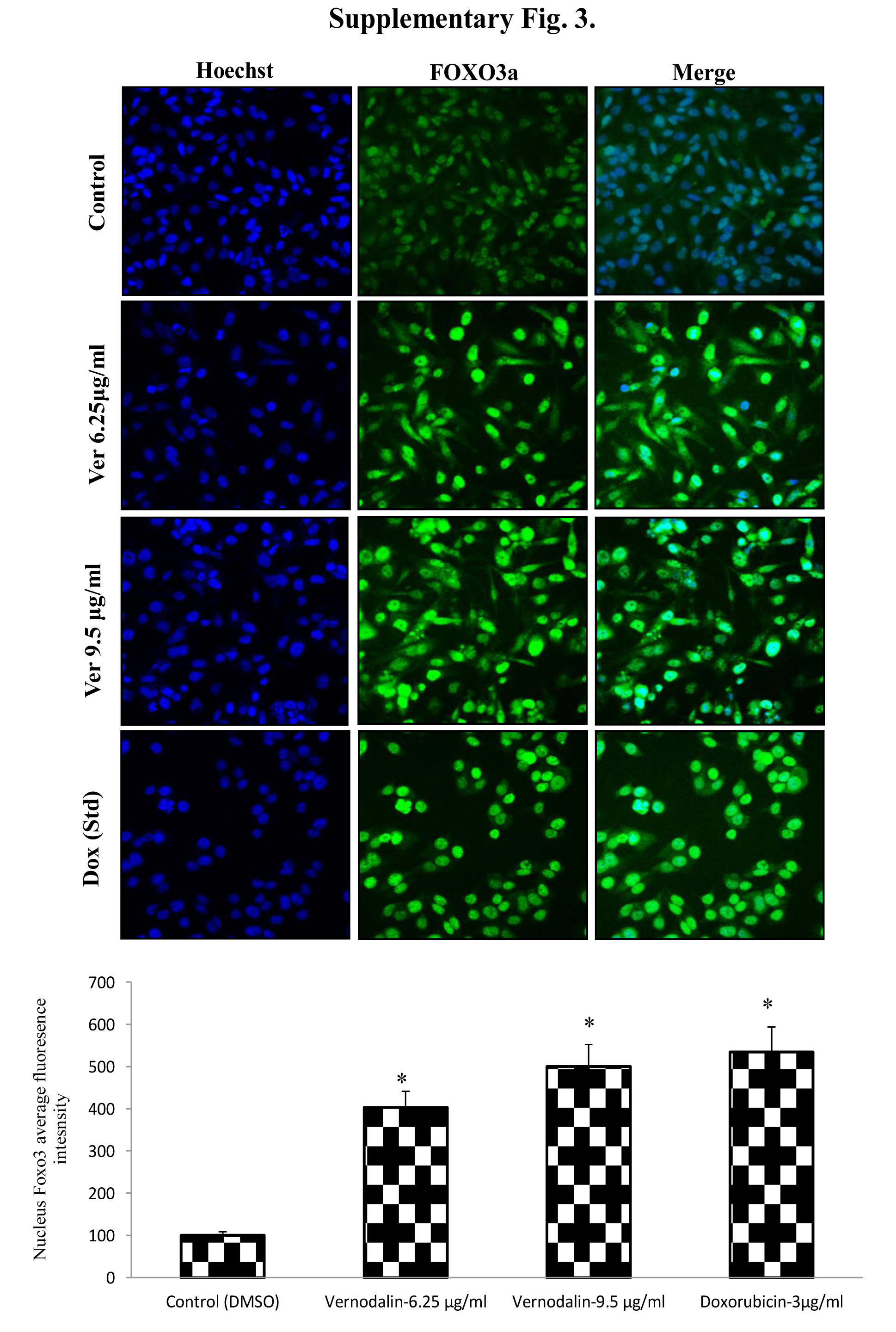

Supplement: Additional file 3: Figure S3. — Vernodalin treatment induces FOXO3a nuclear translocation in MDA-MB-231 cells. MDA-MB231 cells were treated with DMSO (control) or indicated concentration of vernodalin for 24 h. Immunofluorescent staining was then performed using the FOXO3a antibody (green) and stained with Hoechst 33258 (blue). Images were acquired using Cellomic HCS array scan reader (objective 20X). Representative figures (control, 6.25, 9.5 μg/ml of vernodalin and Doxorubicin 3 μg/ml) were shown. Bar chart shows average fluorescence intensities of FOXO3a accumulation in the nucleus. Data were mean ± SD of fluorescence intensity readings representative of three independent experiments. (*P < 0.05). (JPG 573 kb) [file 13046_2015_266_MOESM3_ESM.jpg]

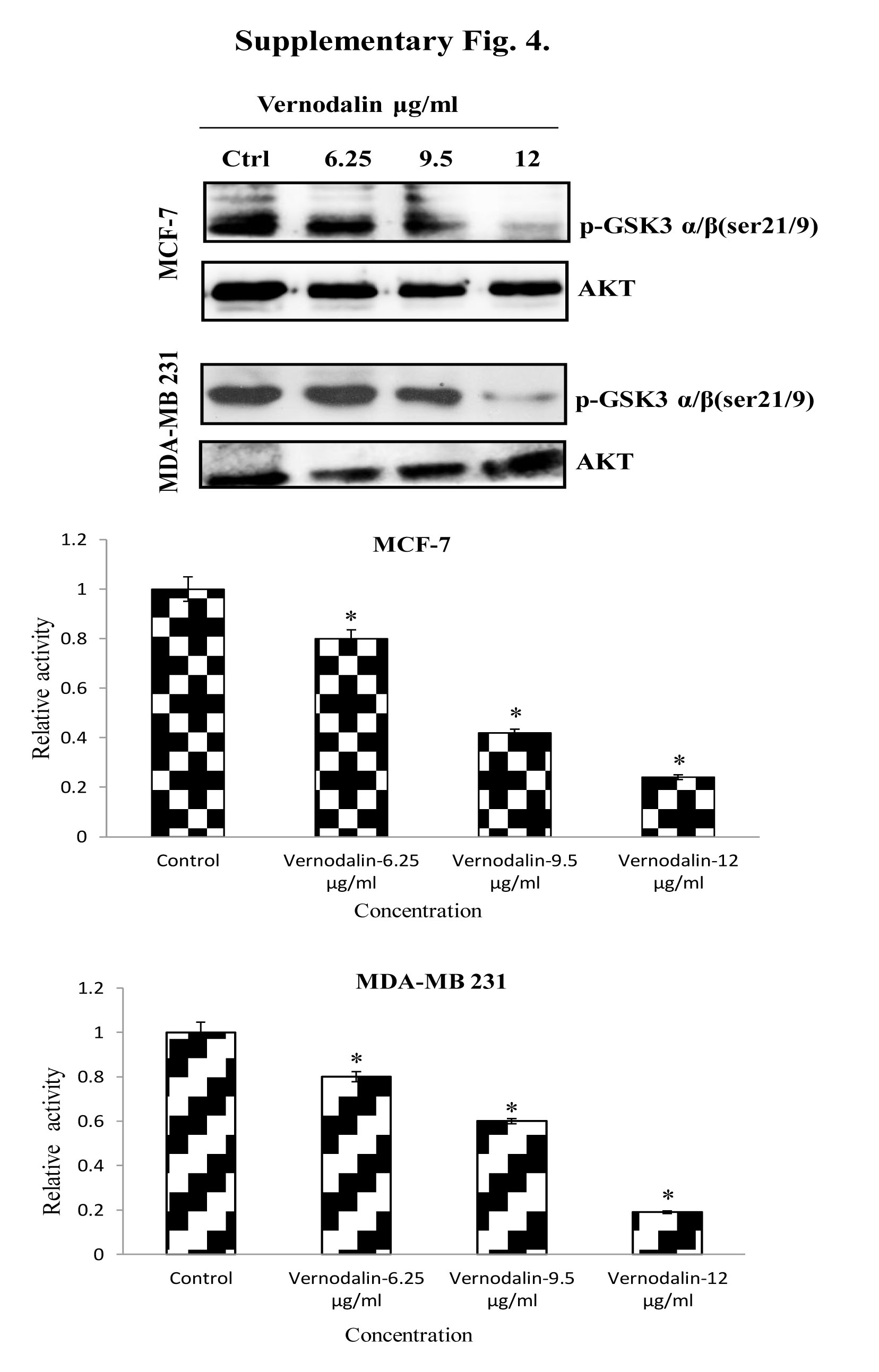

Supplement: Additional file 4: Figure S4. — AKT kinase assay. Akt enzymatic activity was evaluated by phosphorylation of a GSK-3 fusion protein after immunoprecipitation of Akt using a nonradioactive Akt kinase kit as described in the Materials and Methods section. The relative intensity of the p-GSK3α/β band reflected the AKT kinase activity and Total-AKT was immunoblotted as controls. GSK-3 phosphorylation bands were analysed densitometrically. All the treatment groups were compared with control. “*” denotes statistically significant at P < 0.05. Results are shown as percentage values ± SD of three independent experiments. (JPG 318 kb) [file 13046_2015_266_MOESM4_ESM.jpg]

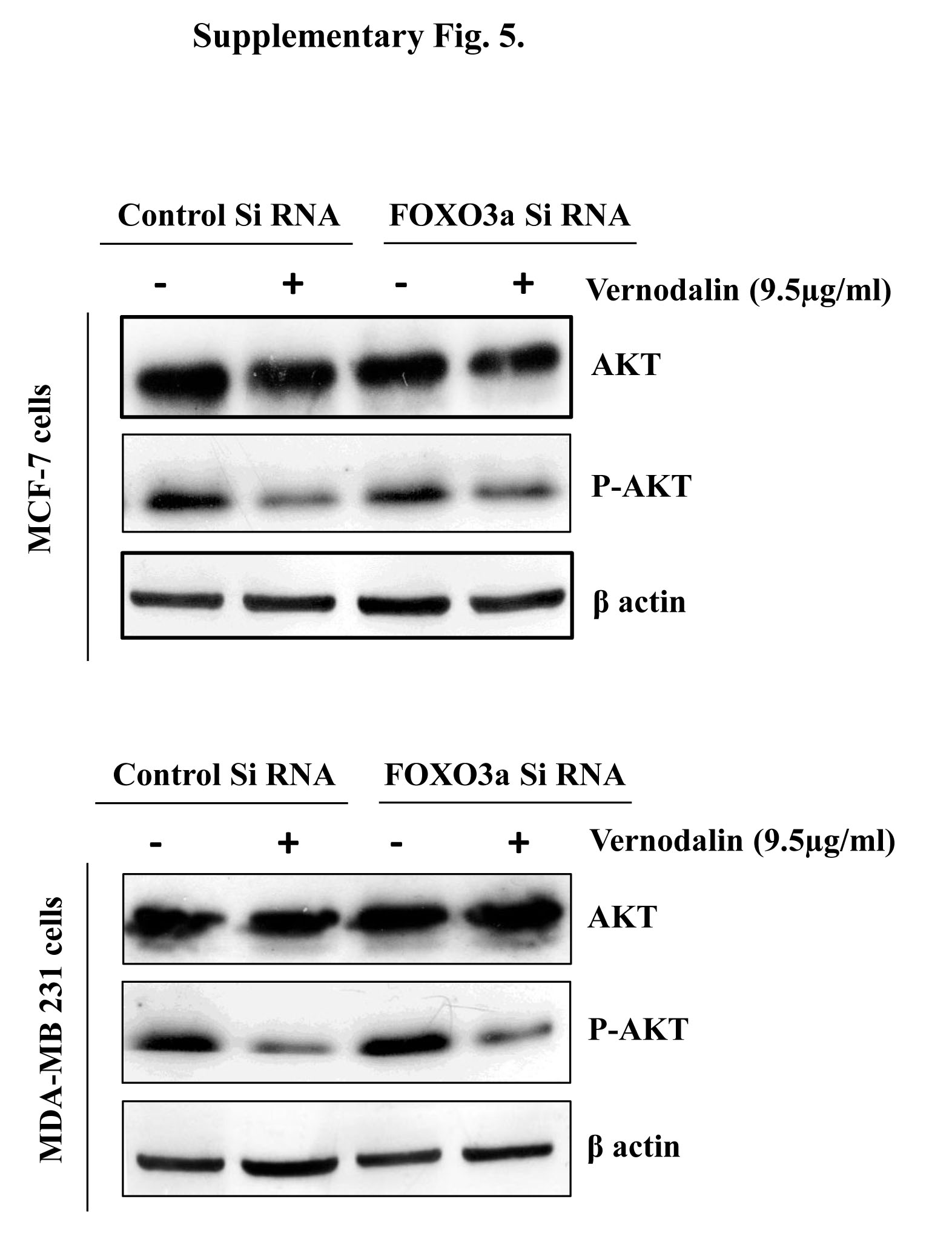

Supplement: Additional file 5: Figure S5. — Effect of FOXO3a-specific siRNA on expression of AKT and p-AKT in response to vernodalin treatment in MCF-7 and MDA-MB231 cells. Cells were pre-treated with scrambled siRNA or siFOXO3a for 48 h, followed by vernodalin (9.5 μg/ml) treatment for 24 h in MCF-7 and MDA-MB231 cells. Protein lysates were prepared at the times indicated and the levels of AKT and p-AKT were analysed by using Western blots. Data are presented as means ± SD of three independent experiments. (JPG 255 kb) [file 13046_2015_266_MOESM5_ESM.jpg]

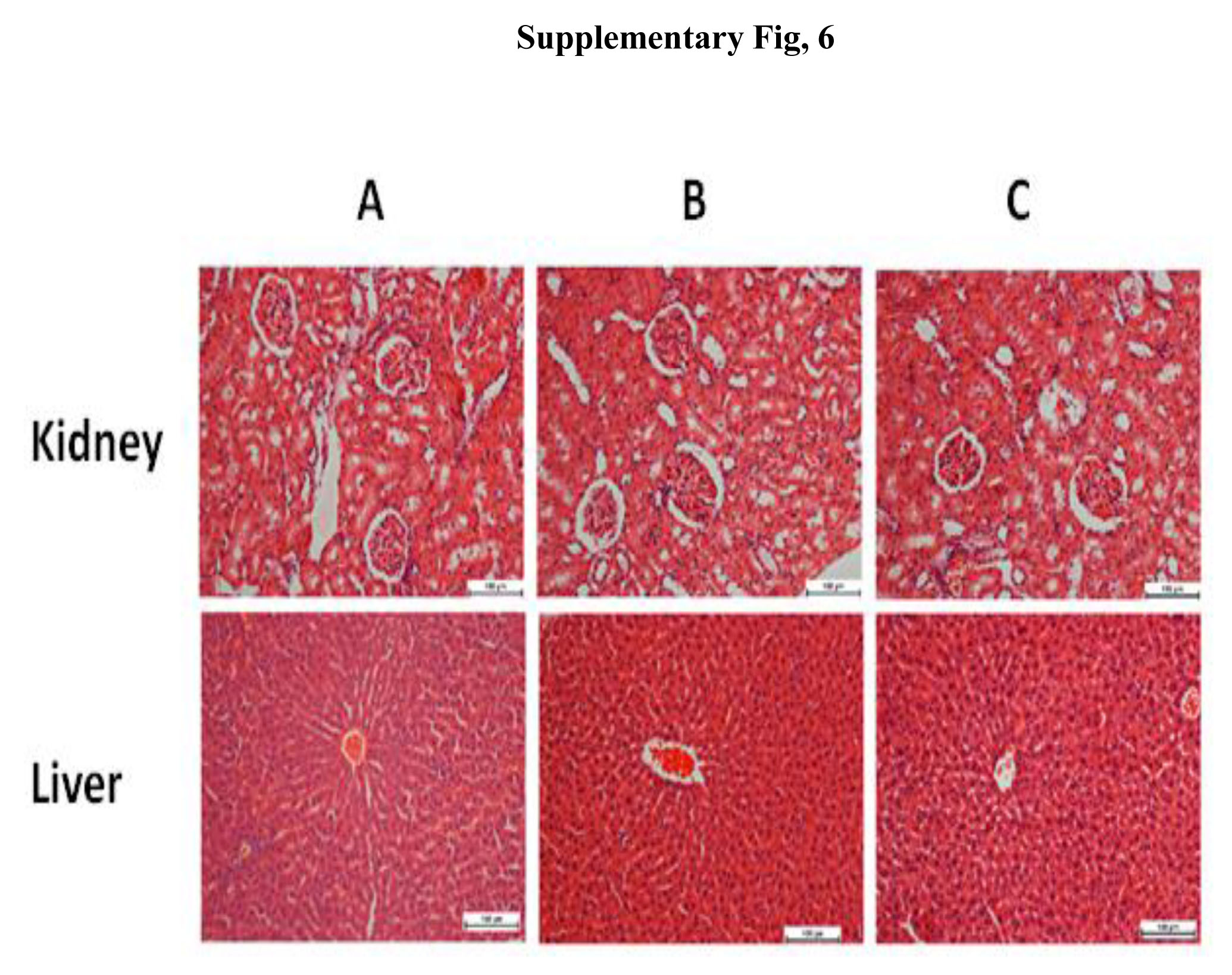

Supplement: Additional file 6: Figure S6. — Histopathological study of kidney and liver tissues from control or vernodalin treated rats. A.control (untreated). B. vernodalin 1 mg/kg- treated. C. vernodalin 10 mg/kg- treated. (n = 5). Liver sections of control and vernodalin treated rats showing normal architecture of hepatocytes with visible central vein and normal arrangement of hepatocytes. Kidney sections of control and vernodalin treated rats showing normal glomerular, tubular sections. Representative micrographs of histologic sections were shown at 20× magnification. (JPG 396 kb) [file 13046_2015_266_MOESM6_ESM.jpg]
